# Accurate prediction of protein secondary structure and solvent accessibility by consensus combiners of sequence and structure information

**DOI:** 10.1186/1471-2105-8-201

**Published:** 2007-06-14

**Authors:** Gianluca Pollastri, Alberto JM Martin, Catherine Mooney, Alessandro Vullo

**Affiliations:** 1 Complex and Adaptive Systems Laboratory, School of Computer Science and Informatics, University College Dublin, Belfield, Dublin 4, Ireland

## Abstract

**Background:**

Structural properties of proteins such as secondary structure and solvent accessibility contribute to three-dimensional structure prediction, not only in the *ab initio *case but also when homology information to known structures is available. Structural properties are also routinely used in protein analysis even when homology is available, largely because homology modelling is lower throughput than, say, secondary structure prediction. Nonetheless, predictors of secondary structure and solvent accessibility are virtually always *ab initio*.

**Results:**

Here we develop high-throughput machine learning systems for the prediction of protein secondary structure and solvent accessibility that exploit homology to proteins of known structure, where available, in the form of simple structural frequency profiles extracted from sets of PDB templates. We compare these systems to their state-of-the-art *ab initio *counterparts, and with a number of baselines in which secondary structures and solvent accessibilities are extracted directly from the templates. We show that structural information from templates greatly improves secondary structure and solvent accessibility prediction quality, and that, on average, the systems significantly enrich the information contained in the templates. For sequence similarity exceeding 30%, secondary structure prediction quality is approximately 90%, close to its theoretical maximum, and 2-class solvent accessibility roughly 85%. Gains are robust with respect to template selection noise, and significant for marginal sequence similarity and for short alignments, supporting the claim that these improved predictions may prove beneficial beyond the case in which clear homology is available.

**Conclusion:**

The predictive system are publicly available at the address .

## Background

Protein secondary structure and solvent accessibility predictions are an important stage towards the prediction of protein structure and function. Accurate secondary structure and solvent accessibility information is not only at the core of most *ab initio *methods for the prediction of protein structure (e.g. see [2]) but is also effective in improving the sensitivity of fold recognition methods (e.g. [3-5]), and is routinely used in protein analysis and annotation [6].

Virtually all modern methods for the prediction of protein one-dimensional structural features (i.e. those features which may be represented as a string of the same length as the primary sequence, such as secondary structure and solvent accessibility) are based on machine learning techniques [7-22], and exploit evolutionary information in the form of amino acid frequency profiles extracted from alignments of multiple sequences, generally of unknown structure. The progress of these methods over the last 10 years has been slow, but steady, and is due to numerous factors: the ever-increasing size of training sets; more sensitive methods for the detection of homologues, such as PSI-BLAST [23]; the use of ensembles of multiple predictors trained independently, sometimes tens of them [12]; more sophisticated machine learning techniques (e.g. [14]); a combination of a number of the above [19].

Predictors of secondary structure and solvent accessibility are virtually always *ab initio *(with very few exceptions, e.g., recently, [22]), meaning that they do not rely directly on similarity to proteins of known structure. In fact, often, much care is taken to try to exclude any detectable similarity between training and test set instances when gauging predictive performances of structural feature predictors. The main reason for this seems to be a short-circuit, which happened early on in the field and was never disputed, between the idea of hypothesis validation by strict training and test set separation (borrowed from statistical learning), and the concept of *ab initio *prediction. For training and test sets to be strictly distinct, they are required to not only contain different examples (which is all the statistical learning principle dictates, together with independence and identical distribution), but to contain examples that do not show significant sequence identity to one another, as detected by a standard BLAST [23] search. A hint of the historical, more than scientific, nature of this issue is the fact that when subtler algorithms for sequence similarity detection became available (e.g. PSI-BLAST [23]), the criteria for training vs. test set separation did not always change.

Currently over half of all known protein sequences show some detectable degree of similarity to one or more sequences of known structure. Nearly 3/4 of newly deposited structures in the PDB [24] show significant similarity to previously deposited structures [22]. Over 60% of the queries received by the server Porter [19] in the first six months of year 2006 have potential homologues in the PDB at the moment of submission (PSI-BLAST e-value smaller than 0.01), and another 25% have marginal similarity to some sequence in the PDB (PSI-BLAST e-value between 0.01 and 10). For the case of clear homology, direct structural information from the homologous proteins can be exploited for the prediction of structural features. For instance, secondary structure extracted from full three-dimensional comparative models is known to be significantly more reliable than secondary structure obtained from *ab initio *predictors [8,22]. Moreover, even where alignments to PDB structures are of dubious reliability, or too short to reliably imply homology, these may carry information. One of the main sources of improvement for fold recognition and *ab initio *structure prediction methods over the last few CASP competitions [25-28] has been the reliance on sets of possible conformations for short fragments of chain [28], extracted from the PDB.

There is a number of reasons why direct, machine learning-based predictions of secondary structure or other structural features incorporating homology information are useful: nearly all the most reliable public predictors [6,9,14,29,30] ([22] is an exception, potentially equally reliable, although currently not tested by independent assessors such as EVA [31]) do not take structural information directly into account, which implies that over half of the responses provided to users could be improved, often dramatically; machine learning methods are robust with respect to noise – selecting a template from a set of candidate structures from the PDB may be less of a problem than in traditional comparative modelling, since a set or a profile of templates (possibly conflicting) may be provided to the method, rather than a single template which might be erroneous; machine learning methods are significantly faster than full comparative modelling methods – large-scale predictions may be generated with relatively modest computational resources, and feed into structure-based functional similarity algorithms, comparative modelling validation and template selection, protein analysis and proteome annotation efforts; low-similarity, short-alignment based predictions may improve on traditional *ab initio *ones in fold recognition or even novel fold cases.

Here we develop high-throughput systems for the prediction of protein secondary structure and solvent accessibility, which exploit similarity to proteins of known structure, where available, in the form of simple structural frequency profiles from sets of PDB templates. The systems have two stages: one in which a set of templates for a query sequence is generated based on a similarity search of the PDB; one in which this template information, plus the primary sequence, and evolutionary information in the form of multiple alignments is used as input to an ensemble of recursive neural networks to determine a query's secondary structure and solvent accessibility. Although here we use a simple PSI-BLAST-based protocol to find suitable templates (see Methods section), our systems are fully modular and may easily accommodate more sophisticated stages with better sensitivity to remote homology (e.g. [5,32]). It is important to stress that, when homology information is available, the systems we design here do not simply take it as the final answer, but rather use it as a further input. This, on average, leads to significant improvements over extracting secondary structure and solvent accessibility directly from the best single PDB template, and weighed and unweighed averages of the top 10 templates, or all templates identified, suggesting that the combination of sequence and template information carries more information than templates alone. Not surprisingly, when only very high quality templates are available (PSI-BLAST e-value smaller than roughly 10^-30^), which are almost guaranteed to be close homologues, the improvements become marginal.

We also compare the predictive systems to to their state-of-the-art *ab initio *counterparts. We show that similarity information, when available, greatly improves prediction quality. For sequence similarity exceeding 30%, prediction quality is nearly at its theoretical maximum. Gains are significant for low sequence similarity when we design specialised systems for this case, and for alignments shorter than 20 residues, outside traditional comparative modelling territory, supporting the claim that these improved predictions may prove beneficial for fold recognition algorithms.

The predictive systems described in this paper are publicly available at the address [1], as part of a suite of predictors of protein structural features. When the user requests secondary structure (Porter) or solvent accessibility (PaleAle) predictions, homology-based results are automatically selected when suitable templates are available. Up to 20,000 queries a day may be served by the 40 CPU cluster hosting the predictors.

## Results and Discussion

The four systems we describe here are: an *ab initio *secondary structure predictor (Porter [19]) in three classes; the same, but homology-based (Porter_H); an *ab initio *predictor of relative solvent accessibility in 4 and 2 classes (PaleAle); the same, but homology-based (PaleAle_H). All systems are trained and tested in rigorous 5-fold cross-validation on the December 2003 25% pdb_select list [33] (for details, see Methods section). We use this set to make direct comparisons with Porter's published results, and performances as recorded by EVA [31]. The public versions of the servers will undergo regular trainings to keep them up-to-date with the expansion of the PDB.

**Figure 1:**
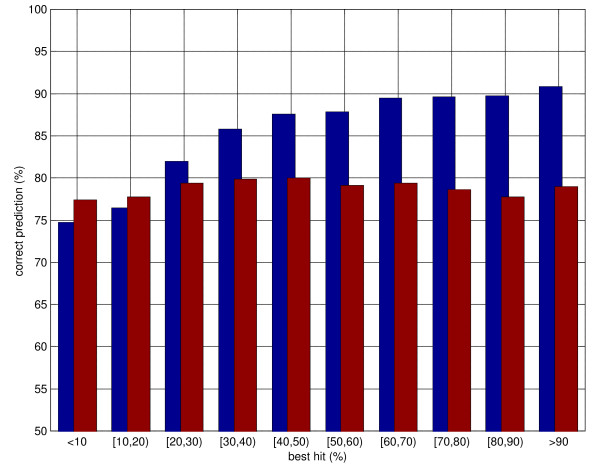
Distribution of secondary structure prediction accuracy as a function of sequence similarity to the best hit in PSI-BLAST templates. The blue bars represent predictions using templates (maximal sequence similarity allowed is 95%), the red bars template-less predictions (Porter). See text for details.

Porter classifies correctly 79.0% of all residues. Porter has currently the highest performance of all predictors tested by independent assessor EVA [31], and is ranked first of all methods by EVA based on the combination of pairwise comparisons of servers on identical sets. Overall, Porter_H classifies correctly 85.7% residues, or nearly 7% above Porter. If we consider only those residues for which PDB template information is available (270,110 out of 344,653), Porter_H's performance rises to 88.3%, roughly 9% above Porter. If we further restrict our observation to those residues for which template information is available with a BLAST e-value of 0.01 or smaller (250,247 residues), Porter_H's performance rises to 89.3% (9.5% above Porter). It is worth reminding that the theoretical maximum for secondary structure prediction performance is well below 100% and is bounded by the intrinsic ambiguity of mapping three-dimensional atom coordinates into secondary structure classes. Any two automated programmes for secondary structure assignment (e.g. [34,35]) differ on at least 5% residues, with up to 20% residues assigned to different states in some cases [36]. Nonetheless, these larger margins are likely mainly due to different definitions of secondary structures by different automated assignment programs, and only by a smaller amount to actual uncertainties as to what the structure might be. Once a semantics is chosen (e.g., as in our case, DSSP) it is possible to classify secondary structure with an accuracy of more than 90% [37]. Hence the theoretical maximum for classification accuracy (i.e. classifying as well as an algorithm which takes as input the experimental structure) is likely somewhere in the 90–95% region. As shown in figure 1, about 90% residues are correctly classified by Porter_H in the 50%-100% similarity (percent identity) region. Nearly 87% are correctly classified between 30 and 50% similarity. Even in the 20–30% similarity region, Porter_H significantly outperforms Porter (82% average classification performance vs. 79%, with standard deviations of 0.3%). For similarity below 20% Porter_H performs slightly worse than Porter. The two main reasons for this are probably: the better specialisation of the latter system, which is trained on 2171 template-less examples, while the former is trained only on about 500 examples with no or very low quality templates; the fact that PSI-BLAST is increasingly inaccurate as sequence similarity becomes lower and noise in the templates eventually dominates over signal.

### Low-similarity templates

**Figure 2:**
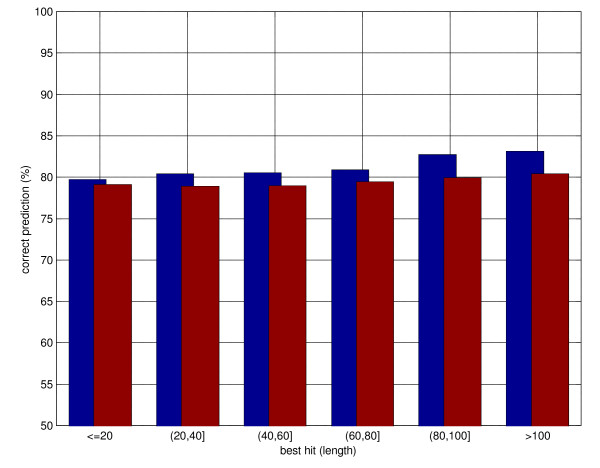
Distribution of secondary structure prediction accuracy as a function of the length of the best hit in PSI-BLAST templates. Maximal 30% identity between template and query allowed. The blue bars represent predictions using templates, the red bars template-less predictions. See text for details.

**Figure 3:**
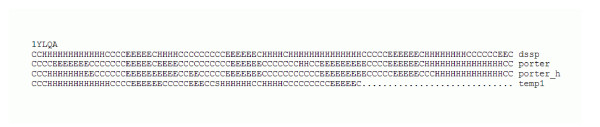
An example of prediction by Porter_H compared to Porter, DSSP assignments, and best template. Best template sequence similarity is 22%. Porter_H correctly identifies the first helix (from the template – strand in Porter), but does not follow the template and assigns correctly the second strand (helix in the template).

To investigate whether a specialised system can yield improvements when only low-similarity templates are available, we re-trained Porter_H twice, with the further constraint that only templates with at most 30% and 20% sequence similarity are adopted, i.e. all PDB templates showing more than 30% (resp. 20%) sequence similarity to the query are eliminated. We refer to the system with maximum 30% similarity templates as Porter_H30 and with maximum 20% similarity templates as Porter_H20. The constraint imposed on template quality implies that many more examples are provided for which no template or only marginal templates are available. Porter_H30's performances in the 0–30% similarity range increase and improve over Porter for all template lengths (see figure 2). Porter_H30 improves consistently over Porter for sequence similarity greater than 17%. Porter_H20 improves consistently over Porter for sequence similarity greater than 13%. Overall, Porter_H30 is not statistically distinguishable from Porter in the 10–20% similarity range, while Porter_H20 actually outperforms Porter in this range. These results suggest that, although noisy, PSI-BLAST-based templates in the 10–20% similarity region still retain information that can be sifted out by a machine learning system, provided that enough examples are available. This also suggests that more subtle similarity tools (e.g. [5,32]) are likely to yield better results in marginal/fold recognition regions if coupled with our predictor. We are in the process of investigating this further point. An example of prediction by Porter_H is reported in Figure 3.

### Porter_H vs. templates

Table 1 reports the comparison between Porter_H and a baseline which simply assigns a residue's secondary structure by copying it from the best template available in the PDB (i.e. that ranked highest by PSI-BLAST). The results are measured only on those residues for which templates exist. Porter_H outperforms the baseline by significant margins for all template qualities allowed: by 2.1% when trained on templates with sequence similarity of up to 95%; by 6.1% for similarity of up to 30%; by 9.3% for similarity of up to 20%.

**Table 1:** Performances of the template-based secondary structure predictor (Porter_H) compared with a baseline predictor which copies the secondary structure of the best template (baseline) and with the *ab initio *secondary structure predictor (*Porter*).

*maxID*	baseline	Porter_H	*Porter*
95%	86.2%	88.3%	*79.6%*
30%	77.6%	83.7%	*80.2%*
20%	73.1%	82.4%	*80.5%*

Templates up to 95%, 30% and 20% maximum similarity allowed (*maxID*) for baseline and Porter_H. Performances measured only on residues for which a template has been identified. Results in 5-fold cross-validation on the S2171 set (see text for details).

We also tested different baselines in which, instead of just the top template, respectively, the top 10 templates (as ranked by PSI-BLAST) and all the templates are used to predict the secondary structure of a protein. In both cases the prediction is obtained as a majority vote among the templates covering each residue. We tested both an unweighed vote (i.e. one in which each template counts the same) and a vote in which each template is weighed by its sequence similarity to the query, cubed. The latter weighing scheme is identical to the one used to present the templates to the network (see Methods section for details), and we refer to it as baseline_input. In all cases the predictions are worse than those obtained by only considering the top template (by at least 2% for the 95% maximum similarity case, and at least 3% for the 30% and 20% maximum similarity cases), hence at least 4% worse than Porter_H.

When we consider templates with sequence similarity of up to 95% and exclude marginal hits (BLAST e-value greater than 0.01), Porter_H still outperforms the baseline by a significant margin, although reduced (0.8%). This continues to be true when the threshold for excluding hits is lowered, down to 10^-40^, beyond which the differences between the best baseline and the predictors become negligible. Table 4 reports the level of disagreement between Porter_H and baseline_input, which is the baseline it agrees most with. Disagreement is measured simply as the percentage of residues on which Porter_H's and baseline_input's predictions are different. The overall disagreement is 9.9%, which decreases to 8.6% on residues for which templates exist with *e *< 0.01, and grows to over 30% when templates with *e *< 0.01 are excluded.

**Table 4:** Percentage of residues on which Porter_H and PaleAle_H disagree with the template-based part of their input (profile of secondary structure/solvent accessibility frequency from templates, in which each template is weighed by its cubed sequence similarity to the query – see Methods section for details).

	Porter_H	PaleAle_H
*e *< 0.01	8.6%	14.4%
*e *> 0.01	30.8%	21.6%
All	9.9%	21.2%

Results on all residues for which a template exists (All), residues for which a template exists with BLAST e-value smaller than 0.01 (*e *< 0.01) and all residues for which a template exists after having removed templates with BLAST e-value smaller than 0.01 (*e *> 0.01). Results in 5-fold cross-validation on the S2171 set (see text for details).

These results suggest that combining sequence and structure information is a better choice than only relying on templates, i.e. the sequence contains enough information to resolve at least some of the ambiguity contained in sets of templates retrieved by sequence similarity.

**Figure 4:**
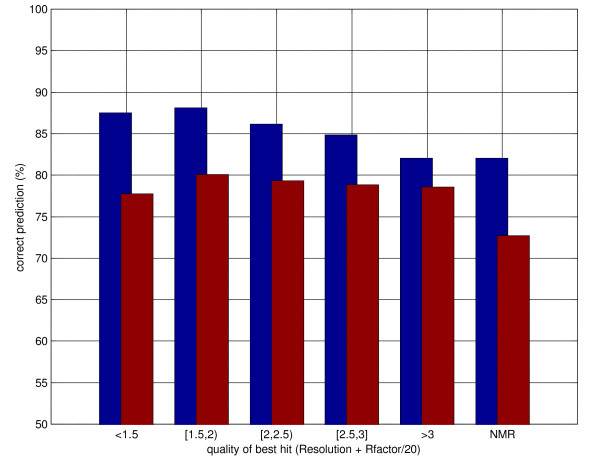
Distribution of secondary structure prediction accuracy as a function of quality of the best hit in PSI-BLAST templates. Quality measured as Resolution+Rfactor/20. The blue bars represent predictions using templates, the red bars template-less predictions (Porter). See text for details.

The distribution of prediction performances as a function of the quality of the best hit (measured as X-ray resolution + R-factor/20) is shown in figure 4. Homology-based predictions are better for all intervals of quality, but not surprisingly the gains decrease with the decrease in quality of the templates. Somewhat surprisingly in the case of NMR templates the gains (but not the overall prediction performances, possibly due to the different distribution of NMR proteins) are comparable to those obtained with high quality templates from X-ray crystallography.

We also checked whether the presence of membrane proteins in the sets we use for training and testing has any influence on the results. In total there are 64 membrane proteins out of 2171 in the set, covering roughly 5% of all amino acids. On these proteins Porter_H outperforms Porter by 5.1% (82.1% correct prediction vs. 77%), less than on the whole set. Removing membrane proteins from the set changes the performances of both Porter and Porter_H by less than 0.1%, and keeps the difference between the methods statistically unchanged.

Lastly, we tested Porter_H on the EVA common2 set as available in November 2004, containing 134 proteins. On this set, a version of Porter retrained from scratch, after having excluded from its training set all sequences with more than 25% similarity to any sequence in the set, achieves 76.8% correct prediction, better by at least 1.9% than all the other servers evaluated. On the same set, a similarly retrained Porter_H achieves 81.5% correct prediction when templates with more than 95% sequence similarity to the query are ignored. In this set for 68 out of 134 sequences the best template is below 30% sequence similarity and for 44 below 20%.

### Solvent Accessibility prediction

**Figure 5:**
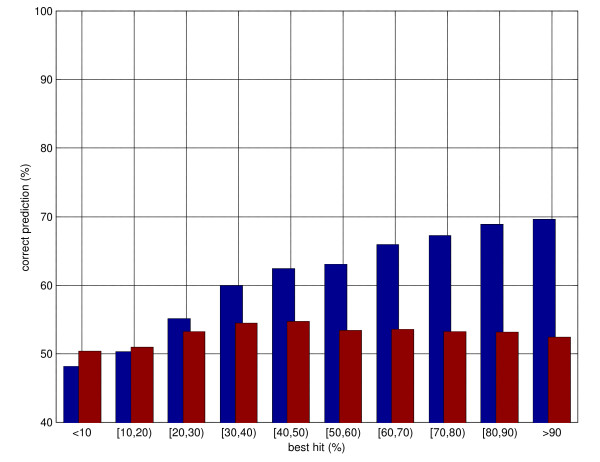
Distribution of 4-class (4%, 25% and 50% exposed thresholds) solvent accessibility prediction accuracy as a function of sequence similarity to the best hit in PSI-BLAST templates. The blue bars represent predictions using templates (maximal sequence similarity allowed is 95%), the red bars template-less predictions. See text for details.

**Figure 6:**
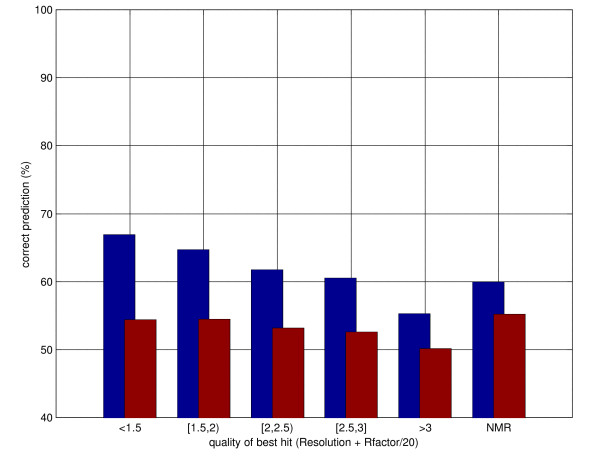
Distribution of 4-class (4%, 25% and 50% exposed thresholds) solvent accessibility prediction accuracy as a function of quality of the best hit in PSI-BLAST templates. Quality measured as Resolution+Rfactor/20. The blue bars represent predictions using templates, the red bars template-less predictions. See text for details.

Similar results are obtained for solvent accessibility prediction, as shown in figures 5 and 6, and table 2. The figures and table refer to 4-class solvent accessibility prediction. The template-less predictor (PaleAle) achieves 53.3% correct 4-class prediction. If the 4 classes are reassigned into 2 with a 25% accessibility threshold (simply by merging the first two classes and the last two classes) the performance rises to 78.9%. Although comparisons on different sets are always not entirely fair, this is at least as good as the most recent 2-class predictors adopting the same class threshold, e.g. [21] (78.1%), [20] (77.7%), [38] (78.5%), [18] (76.7%).

**Table 2:** Performances of the template-based 4-class solvent accessibility predictor (PaleAle_H) compared with a baseline predictor which copies the solvent accessibility of the best template (baseline) and with the *ab initio *solvent accessibility predictor (*PaleAle*).

*maxID*	baseline	PaleAle_H	*PaleAle*
95%	61.1%	64.8%	*53.8%*
30%	49.2%	57.1%	*54.3%*
20%	45.6%	55.5%	*54.4%*

Templates up to 95%, 30% and 20% maximum similarity allowed (*maxID*) for baseline and PaleAle_H. Performances measured only on residues for which a template has been identified. Results in 5-fold cross-validation on the S2171 set (see text for details).

Table 3 reports a comparison of PaleAle on the Manesh set [39], containing 215 proteins, against a number of other predictors (data from [21]). For this test we retrained PaleAle after having excluded from its training set all sequences with over 25% sequence similarity to any sequence in the Manesh set (this reduced the size of the set from 2171 to 1662 proteins). After retraining, PaleAle achieves 79.2% correct prediction on the set (52.5% in 4 classes), or at least 1.1% better than any of the other predictors tested. The template-based predictor (PaleAle_H) classifies correctly 61.8% of all residues (82.6% for the 2-class problem), gaining significantly for template identity above 20%, and slightly underperforming compared to PaleAle for best template identity below 20%. On the 270,110 residues for which template information is available, the performance grows to 64.8%. On the Manesh set, the 2-class, retrained version of PaleAle_H classifies correctly 86.0% of all residues (68.0% for 4 classes), or 6.8% better than PaleAle (15.5% for 4 classes). Gains and losses are all of the same sign and of similar magnitude as in the secondary structure case, counter to the assumption that solvent accessibility is less clearly conserved than secondary structure, hence harder to fathom from templates. Even in the case of solvent accessibility, gains over a baseline predictor copying the accessibility of the best template are large for all levels of maximal sequence similarity allowed: 3.7% for maximal similarity of 95%; 7.9% for 30%; 9.9% for 20%. In all three cases the template-based predictor outperforms, on average, its *ab initio *variant. For solvent accessibility we also tested different baselines in which the top 10 templates and all templates found by PSI-BLAST are considered, both unweighed and weighed by the cube of their sequence similarity to the query (baseline_input), as in the case of secondary structure. In this case, though, instead of a having a majority vote, for each residue we computed the (weighed or unweighed) average of the solvent accessibility values from the templates and then used this average to determine the solvent accessibility class. As in the secondary structure case, these baselines yielded lower performances than the one based on the top template, by at least 1% for the 95% maximum similarity case, and 2% for both the 30% and 20% maximum similarity case, or always at least 3% worse than PaleAle_H. When we focus only on more reliable templates (PSI-BLAST e-value smaller than 0.01) PaleAle_H still outperforms all the baselines by significant margins (at least 1.2%) on the residues covered, and disagrees on 14.4% of residues with its closest baseline (baseline_input. See Table 4). When we tighten the e-value threshold to exclude hits from 0.01 down to *e *< 10^-20^, PaleAle_H still significantly outperforms all the baselines. Beyond *e *< 10^-20 ^PaleAle_H's improvements over the baselines become marginal. As in the case of secondary structure, removing membrane proteins from the sets leaves the results statistically unchanged.

**Table 3:** Performances of the two-class PaleAle and PaleAle_H compared with a number of recent methods on the Manesh dataset [39].

Method	
PaleAle_H	86.0%
PaleAle	79.2%
NETASA [15]	70.3%
[21]	78.1%
PP [47]	78.1%
PredAcc [11]	70.7%
JNET [13]	75.0%
ACCpro [16]	77.2%
SABLE [17]	77.6%

Performances of the various methods from [21]. The class threshold is 25% for all methods. Templates up to 95% adopted by PaleAle_H.

## Conclusion

We have developed high-throughput systems for the prediction of protein secondary structure and solvent accessibility, exploiting similarity to proteins of known structure. These systems, based on machine learning techniques, greatly outperform their *ab initio *counterparts when PDB templates are available, are capable of combining sequence information and structural information from multiple templates, and outperform simpler strategies such as the extraction of the structural properties in question from the best available template in the PDB, or from weighed and unweighed profiles of templates. Moreover, they are entirely automated, and can be run on multi-genomic or bioengineering scales. On a small cluster of machines, hundreds of thousands of protein structural features may be predicted in days.

What is especially encouraging is that performance gains are significant even for marginal sequence similarity when we design specialised systems for this case. This suggests that our strategy may feed into fold recognition systems, which currently rely on *ab initio *secondary structure predictors. A closed-loop strategy in which the results of fold recognition searches are fed back into the predictors is also possible, and is the object of our current investigation.

All predictive systems are available at the address [1]. Template-based predictions are automatically returned by the secondary structure prediction server (Porter) and the solvent accessibility server (PaleAle) when templates showing more than 20% sequence similarity to the query are detected. Given the current distribution of queries, this will yield greatly improved predictions for well over half of all requests.

## Methods

### Training set

The data set used in our simulations is extracted from the December 2003 25% pdb_select list [33]. We assign each residue's secondary structure and solvent accessibility using the DSSP program [34]. The relative solvent accessibility of a residue is defined as the accessibility in Å^2 ^as computed by DSSP, divided by the maximum observed accessibility for that type of residue. Secondary structure is mapped from the 8 DSSP classes into three classes as follows: H, G, I → Helix; E, B → Strand; S, T,. → Coil. Relative solvent accessibility is mapped into 4 classes where class thresholds are chosen to be maximally informative, i.e. to split the set into (roughly) equally numerous classes: [0%, 4%), [4%, 25%), [25%, 50%) and [50%, ∞) exposed.

We remove all sequences for which DSSP does not produce an output due, for instance, to missing entries (e.g. if only the C_*α *_trace is present in the PDB file) or format errors. After processing by DSSP, this set (S2171) contains 2171 proteins and 344,653 amino acids. All the tests reported in this paper are run in 5-fold cross validation on S2171. The 5 folds are of roughly equal sizes, composed of 434–435 proteins and ranging between 67,345 and 70,098 residues. The datasets are available upon request.

Prediction from a multiple alignment of protein sequences rather than a single sequence has long been recognised as a way to improve prediction accuracy for virtually all protein structural features: secondary structure [9,10,14,19,22,29,40], solvent accessibility [11,13,15-18,20,21], beta-sheet pairing [41,42], contact maps [43-45], etc. We exploit evolutionary information in the form of frequency profiles compiled from alignments of multiple homologous sequences, extracted from the NR database. Multiple sequence alignments for the S2171 set are extracted from the NR database as available on March 3 2004 containing over 1.4 million sequences. The database is first redundancy reduced at a 98% threshold, leading to a final 1.05 million sequences. The alignments are generated by three runs of PSI-BLAST [23] with parameters *b *= 3000 (maximum number of hits), *e *= 10^-3 ^(expectation of a random hit) and *h *= 10^-10 ^(expectation of a random hit for sequences used to generate the PSSM).

Data sets, training/test folds and multiple alignments are identical to those used to train and test the *ab initio *secondary structure predictor Porter [19].

### Template generation

For each of the proteins in S2171 we search for structural templates in the PDB. We base our search on PDBFINDERII [46] as available on August 22 2005. An obvious problem arising is that all proteins in the S2171 set are expected to be in PDB (barring name changes), hence every protein will have a perfect template. To avoid this, we exclude from PDBFINDERII every protein that appears in S2171. We also exclude all entries shorter than 10 residues, leading to a final 66,350 chains. Because of the PDBFINDERII origin, only one chain is present in this set for NMR entries.

To generate the actual templates for a protein, we run two rounds of PSI-BLAST against the version of the redundancy-reduced NR database described above, with parameters *b *= 3000, *e *= 10^-3 ^and *h *= 10^-10^. We then run a third round of PSI-BLAST against the PDB using the PSSM generated in the first two rounds. In this third round we deliberately use a high expectation parameter (*e *= 10) to include hits that are beyond the usual Comparative Modelling scope (*e *< 0.01, at the CASP6 competition [28]). We further remove from each set of hits thus found all those with sequence similarity exceeding 95% over the whole query, to exclude PDB resubmissions of the same structure at different resolution, other chains in N-mers and close homologues.

**Figure 7:**
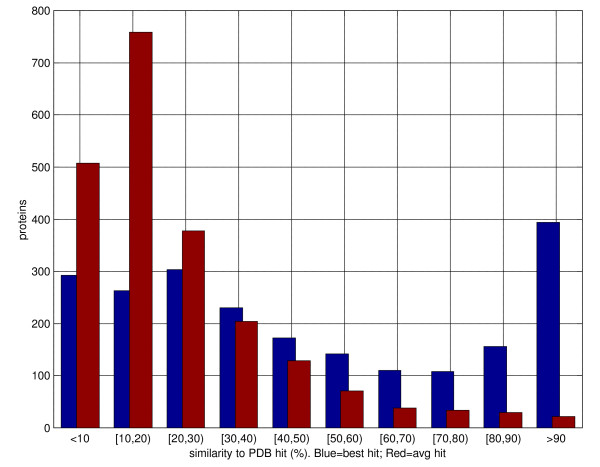
Distribution of best-hit (blue) and average (red) sequence similarity in the PSI-BLAST templates for the S2171 set. Hits above 95% sequence similarity excluded.

The distribution of sequence similarity of the best template, and average template similarity is plotted in figure 7. Roughly 15% of the proteins have no hits at more than 10% sequence similarity. About 20% of all proteins have at least one very high quality (better than 90% similarity) entry in their template set.

Although the distribution is not uniform, all similarity intervals are adequately represented: for about 40% of the proteins no hit is above 30% similarity; for nearly 20% of the proteins the best hit is in the 30–50% similarity interval. Overall 74,543 residues (21.6% of the set) are not covered by any template. The average similarity for all PDB hits for each protein, not surprisingly, is generally low: for roughly 75% of all proteins in S2171 the average identity is below 30%.

To test template-based predictions in marginal similarity conditions we also extract two further template sets from which all hits are excluded that exceed, respectively, 30% and 20% sequence similarity. In this case the number of residues not covered by any template climbs, respectively, to 148,124 (43% of the total) and 193,921 (56.3%).

### Predictive architectures

To learn the mapping between our input space ℐ
 MathType@MTEF@5@5@+=feaafiart1ev1aaatCvAUfKttLearuWrP9MDH5MBPbIqV92AaeXatLxBI9gBamrtHrhAL1wy0L2yHvtyaeHbnfgDOvwBHrxAJfwnaebbnrfifHhDYfgasaacH8akY=wiFfYdH8Gipec8Eeeu0xXdbba9frFj0=OqFfea0dXdd9vqai=hGuQ8kuc9pgc9s8qqaq=dirpe0xb9q8qiLsFr0=vr0=vr0dc8meaabaqaciaacaGaaeqabaWaaeGaeaaakeaaimaacqWFqessaaa@3769@ and output space O
 MathType@MTEF@5@5@+=feaafiart1ev1aaatCvAUfKttLearuWrP9MDH5MBPbIqV92AaeXatLxBI9gBamrtHrhAL1wy0L2yHvtyaeHbnfgDOvwBHrxAJfwnaebbnrfifHhDYfgasaacH8akY=wiFfYdH8Gipec8Eeeu0xXdbba9frFj0=OqFfea0dXdd9vqai=hGuQ8kuc9pgc9s8qqaq=dirpe0xb9q8qiLsFr0=vr0=vr0dc8meaabaqaciaacaGaaeqabaWaaeGaeaaakeaaimaacqWFoe=taaa@383D@ we use two-layered architectures composed of Bidirectional Recurrent Neural Networks (BRNN)(Also known as 1D-RNN, e.g. in [44]) [10] of the same length *N *as the amino acid sequence. Similarly to [19] we use BRNNs with shortcut connections. In these BRNNs, connections along the forward and backward hidden chains span more than 1-residue intervals, creating shorter paths between inputs and outputs. These networks take the form:

oj=N(O)(ij,hj(F),hj(B))hj(F)=N(F)(ij,hj−1(F),...,hj−S(F))hj(B)=N(B)(ij,hj+1(B),...,hj+S(B))j=1,...,N
 MathType@MTEF@5@5@+=feaafiart1ev1aaatCvAUfKttLearuWrP9MDH5MBPbIqV92AaeXatLxBI9gBaebbnrfifHhDYfgasaacH8akY=wiFfYdH8Gipec8Eeeu0xXdbba9frFj0=OqFfea0dXdd9vqai=hGuQ8kuc9pgc9s8qqaq=dirpe0xb9q8qiLsFr0=vr0=vr0dc8meaabaqaciaacaGaaeqabaqabeGadaaakeaafaqabeabdaaaaeaacqWGVbWBdaWgaaWcbaGaemOAaOgabeaaaOqaaiabg2da9aqaamrtHrhAL1wy0L2yHvtyaeHbnfgDOvwBHrxAJfwnaGabaiab=1q8onaaCaaaleqabaGaeiikaGIaem4ta8KaeiykaKcaaOWaaeWaaeaacqWGPbqAdaWgaaWcbaGaemOAaOgabeaakiabcYcaSiabdIgaOnaaDaaaleaacqWGQbGAaeaacqGGOaakcqWGgbGrcqGGPaqkaaGccqGGSaalcqWGObaAdaqhaaWcbaGaemOAaOgabaGaeiikaGIaemOqaiKaeiykaKcaaaGccaGLOaGaayzkaaaabaGaemiAaG2aa0baaSqaaiabdQgaQbqaaiabcIcaOiabdAeagjabcMcaPaaaaOqaaiabg2da9aqaaiab=1q8onaaCaaaleqabaGaeiikaGIaemOrayKaeiykaKcaaOWaaeWaaeaacqWGPbqAdaWgaaWcbaGaemOAaOgabeaakiabcYcaSiabdIgaOnaaDaaaleaacqWGQbGAcqGHsislcqaIXaqmaeaacqGGOaakcqWGgbGrcqGGPaqkaaGccqGGSaalcqGGUaGlcqGGUaGlcqGGUaGlcqGGSaalcqWGObaAdaqhaaWcbaGaemOAaOMaeyOeI0Iaem4uamfabaGaeiikaGIaemOrayKaeiykaKcaaaGccaGLOaGaayzkaaaabaGaemiAaG2aa0baaSqaaiabdQgaQbqaaiabcIcaOiabdkeacjabcMcaPaaaaOqaaiabg2da9aqaaiab=1q8onaaCaaaleqabaGaeiikaGIaemOqaiKaeiykaKcaaOWaaeWaaeaacqWGPbqAdaWgaaWcbaGaemOAaOgabeaakiabcYcaSiabdIgaOnaaDaaaleaacqWGQbGAcqGHRaWkcqaIXaqmaeaacqGGOaakcqWGcbGqcqGGPaqkaaGccqGGSaalcqGGUaGlcqGGUaGlcqGGUaGlcqGGSaalcqWGObaAdaqhaaWcbaGaemOAaOMaey4kaSIaem4uamfabaGaeiikaGIaemOqaiKaeiykaKcaaaGccaGLOaGaayzkaaaabaaabaaabaGaemOAaOMaeyypa0JaeGymaeJaeiilaWIaeiOla4IaeiOla4IaeiOla4IaeiilaWIaemOta4eaaaaa@A304@

where *i*_*j *_(resp. *o*_*j*_) is the input (resp. output) of the network in position *j*, and hj(F)
 MathType@MTEF@5@5@+=feaafiart1ev1aaatCvAUfKttLearuWrP9MDH5MBPbIqV92AaeXatLxBI9gBaebbnrfifHhDYfgasaacH8akY=wiFfYdH8Gipec8Eeeu0xXdbba9frFj0=OqFfea0dXdd9vqai=hGuQ8kuc9pgc9s8qqaq=dirpe0xb9q8qiLsFr0=vr0=vr0dc8meaabaqaciaacaGaaeqabaqabeGadaaakeaacqWGObaAdaqhaaWcbaGaemOAaOgabaGaeiikaGIaemOrayKaeiykaKcaaaaa@3256@ and hj(B)
 MathType@MTEF@5@5@+=feaafiart1ev1aaatCvAUfKttLearuWrP9MDH5MBPbIqV92AaeXatLxBI9gBaebbnrfifHhDYfgasaacH8akY=wiFfYdH8Gipec8Eeeu0xXdbba9frFj0=OqFfea0dXdd9vqai=hGuQ8kuc9pgc9s8qqaq=dirpe0xb9q8qiLsFr0=vr0=vr0dc8meaabaqaciaacaGaaeqabaqabeGadaaakeaacqWGObaAdaqhaaWcbaGaemOAaOgabaGaeiikaGIaemOqaiKaeiykaKcaaaaa@324E@ are forward and backward chains of hidden vectors with h0(F)=hN+1(B)=0
 MathType@MTEF@5@5@+=feaafiart1ev1aaatCvAUfKttLearuWrP9MDH5MBPbIqV92AaeXatLxBI9gBaebbnrfifHhDYfgasaacH8akY=wiFfYdH8Gipec8Eeeu0xXdbba9frFj0=OqFfea0dXdd9vqai=hGuQ8kuc9pgc9s8qqaq=dirpe0xb9q8qiLsFr0=vr0=vr0dc8meaabaqaciaacaGaaeqabaqabeGadaaakeaacqWGObaAdaqhaaWcbaGaeGimaadabaGaeiikaGIaemOrayKaeiykaKcaaOGaeyypa0JaemiAaG2aa0baaSqaaiabd6eaojabgUcaRiabigdaXaqaaiabcIcaOiabdkeacjabcMcaPaaakiabg2da9iabicdaWaaa@3C31@. We parametrise the output update, forward update and backward update functions (respectively N
 MathType@MTEF@5@5@+=feaafiart1ev1aaatCvAUfKttLearuWrP9MDH5MBPbIqV92AaeXatLxBI9gBamrtHrhAL1wy0L2yHvtyaeHbnfgDOvwBHrxAJfwnaebbnrfifHhDYfgasaacH8akY=wiFfYdH8Gipec8Eeeu0xXdbba9frFj0=OqFfea0dXdd9vqai=hGuQ8kuc9pgc9s8qqaq=dirpe0xb9q8qiLsFr0=vr0=vr0dc8meaabaqaciaacaGaaeqabaWaaeGaeaaakeaaimaacqWFneVtaaa@383B@^(*O*)^, N
 MathType@MTEF@5@5@+=feaafiart1ev1aaatCvAUfKttLearuWrP9MDH5MBPbIqV92AaeXatLxBI9gBamrtHrhAL1wy0L2yHvtyaeHbnfgDOvwBHrxAJfwnaebbnrfifHhDYfgasaacH8akY=wiFfYdH8Gipec8Eeeu0xXdbba9frFj0=OqFfea0dXdd9vqai=hGuQ8kuc9pgc9s8qqaq=dirpe0xb9q8qiLsFr0=vr0=vr0dc8meaabaqaciaacaGaaeqabaWaaeGaeaaakeaaimaacqWFneVtaaa@383B@^(*F*) ^and N
 MathType@MTEF@5@5@+=feaafiart1ev1aaatCvAUfKttLearuWrP9MDH5MBPbIqV92AaeXatLxBI9gBamrtHrhAL1wy0L2yHvtyaeHbnfgDOvwBHrxAJfwnaebbnrfifHhDYfgasaacH8akY=wiFfYdH8Gipec8Eeeu0xXdbba9frFj0=OqFfea0dXdd9vqai=hGuQ8kuc9pgc9s8qqaq=dirpe0xb9q8qiLsFr0=vr0=vr0dc8meaabaqaciaacaGaaeqabaWaaeGaeaaakeaaimaacqWFneVtaaa@383B@^(*B*)^) using three two-layered feed-forward neural networks.

#### Encoding sequence and template information

Input *i*_*j *_associated with the *j*-th residue contains primary sequence information and evolutionary information, and direct structural information derived from PDB templates:

ij=(ij(E),ij(T))
 MathType@MTEF@5@5@+=feaafiart1ev1aaatCvAUfKttLearuWrP9MDH5MBPbIqV92AaeXatLxBI9gBaebbnrfifHhDYfgasaacH8akY=wiFfYdH8Gipec8Eeeu0xXdbba9frFj0=OqFfea0dXdd9vqai=hGuQ8kuc9pgc9s8qqaq=dirpe0xb9q8qiLsFr0=vr0=vr0dc8meaabaqaciaacaGaaeqabaqabeGadaaakeaacqWGPbqAdaWgaaWcbaGaemOAaOgabeaakiabg2da9iabcIcaOiabdMgaPnaaDaaaleaacqWGQbGAaeaacqGGOaakcqWGfbqrcqGGPaqkaaGccqGGSaalcqWGPbqAdaqhaaWcbaGaemOAaOgabaGaeiikaGIaemivaqLaeiykaKcaaOGaeiykaKcaaa@3EB8@

where, assuming that *e *units are devoted to sequence and evolutionary information, and *t *to structural information:

ij(E)=(ij,1(E),...,ij,e(E))
 MathType@MTEF@5@5@+=feaafiart1ev1aaatCvAUfKttLearuWrP9MDH5MBPbIqV92AaeXatLxBI9gBaebbnrfifHhDYfgasaacH8akY=wiFfYdH8Gipec8Eeeu0xXdbba9frFj0=OqFfea0dXdd9vqai=hGuQ8kuc9pgc9s8qqaq=dirpe0xb9q8qiLsFr0=vr0=vr0dc8meaabaqaciaacaGaaeqabaqabeGadaaakeaacqWGPbqAdaqhaaWcbaGaemOAaOgabaGaeiikaGIaemyrauKaeiykaKcaaOGaeyypa0JaeiikaGIaemyAaK2aa0baaSqaaiabdQgaQjabcYcaSiabigdaXaqaaiabcIcaOiabdweafjabcMcaPaaakiabcYcaSiabc6caUiabc6caUiabc6caUiabcYcaSiabdMgaPnaaDaaaleaacqWGQbGAcqGGSaalcqWGLbqzaeaacqGGOaakcqWGfbqrcqGGPaqkaaGccqGGPaqkaaa@48EF@

and:

ij(T)=(ij,1(T),ij,t(T))
 MathType@MTEF@5@5@+=feaafiart1ev1aaatCvAUfKttLearuWrP9MDH5MBPbIqV92AaeXatLxBI9gBaebbnrfifHhDYfgasaacH8akY=wiFfYdH8Gipec8Eeeu0xXdbba9frFj0=OqFfea0dXdd9vqai=hGuQ8kuc9pgc9s8qqaq=dirpe0xb9q8qiLsFr0=vr0=vr0dc8meaabaqaciaacaGaaeqabaqabeGadaaakeaacqWGPbqAdaqhaaWcbaGaemOAaOgabaGaeiikaGIaemivaqLaeiykaKcaaOGaeyypa0JaeiikaGIaemyAaK2aa0baaSqaaiabdQgaQjabcYcaSiabigdaXaqaaiabcIcaOiabdsfaujabcMcaPaaakiabcYcaSiabdMgaPnaaDaaaleaacqWGQbGAcqGGSaalcqWG0baDaeaacqGGOaakcqWGubavcqGGPaqkaaGccqGGPaqkaaa@45DB@

Hence *i*_*j *_contains a total of *e *+ *t *components.

As in [19]*e *= 25: beside the 20 standard amino acids, B (aspartic acid or asparagine), U (selenocysteine), X (unknown), Z (glutamic acid or glutamine) and · (gap) are considered. The input presented to the networks is the frequency of each of the 24 non-gap symbols, plus the overall frequency of gaps in each column of the alignment. I.e., if *n*_*jk *_is the total number of occurrences of symbol *j *in column *k*, and *g*_*k *_the number of gaps in the same column, the *j*^*th *^input to the networks in position *k *is:

njk∑v=124nvk
 MathType@MTEF@5@5@+=feaafiart1ev1aaatCvAUfKttLearuWrP9MDH5MBPbIqV92AaeXatLxBI9gBaebbnrfifHhDYfgasaacH8akY=wiFfYdH8Gipec8Eeeu0xXdbba9frFj0=OqFfea0dXdd9vqai=hGuQ8kuc9pgc9s8qqaq=dirpe0xb9q8qiLsFr0=vr0=vr0dc8meaabaqaciaacaGaaeqabaqabeGadaaakeaadaWcaaqaaiabd6gaUnaaBaaaleaacqWGQbGAcqWGRbWAaeqaaaGcbaWaaabmaeaacqWGUbGBdaWgaaWcbaGaemODayNaem4AaSgabeaaaeaacqWG2bGDcqGH9aqpcqaIXaqmaeaacqaIYaGmcqaI0aana0GaeyyeIuoaaaaaaa@3CC2@

for *j *= 1...24, while the 25^*th *^input is:

gkgk+∑v=124nvk
 MathType@MTEF@5@5@+=feaafiart1ev1aaatCvAUfKttLearuWrP9MDH5MBPbIqV92AaeXatLxBI9gBaebbnrfifHhDYfgasaacH8akY=wiFfYdH8Gipec8Eeeu0xXdbba9frFj0=OqFfea0dXdd9vqai=hGuQ8kuc9pgc9s8qqaq=dirpe0xb9q8qiLsFr0=vr0=vr0dc8meaabaqaciaacaGaaeqabaqabeGadaaakeaadaWcaaqaaiabdEgaNnaaBaaaleaacqWGRbWAaeqaaaGcbaGaem4zaC2aaSbaaSqaaiabdUgaRbqabaGccqGHRaWkdaaeWaqaaiabd6gaUnaaBaaaleaacqWG2bGDcqWGRbWAaeqaaaqaaiabdAha2jabg2da9iabigdaXaqaaiabikdaYiabisda0aqdcqGHris5aaaaaaa@3F25@

This input coding scheme is richer than simple 20-letter schemes and has proven effective in [19].

In the case of secondary structure prediction we use *t *= 10 for representing structural information from the templates. Hence the total number of inputs for a given residue is *e *+ *t *= 35. The first 8 structural input units contain the average 8-class (DSSP style) secondary structure composition in the PDB templates, while the last 2 encode the average quality of the template column. Assume that *s*_*p,j *_is an 8-component vector encoding the DSSP-assigned 8-class secondary structure of *j*-th residue in the *p*-th template as follows:

*H *= (1, 0, 0, 0, 0, 0, 0, 0)

*G *= (0, 1, 0, 0, 0, 0, 0, 0)

*I *= (0, 0, 1, 0, 0, 0, 0, 0)

*E *= (0, 0, 0, 1, 0, 0, 0, 0)

*B *= (0, 0, 0, 0, 1, 0, 0, 0)

*S *= (0, 0, 0, 0, 0, 1, 0, 0)

*T *= (0, 0, 0, 0, 0, 0, 1, 0)

· = (0, 0, 0, 0, 0, 0, 0, 1)

Then, if *P *is the total number of templates for a protein:

(ij,1(T),...,ij,8(T))=∑p=1Pwpsp,j∑p=1Pwp
 MathType@MTEF@5@5@+=feaafiart1ev1aaatCvAUfKttLearuWrP9MDH5MBPbIqV92AaeXatLxBI9gBaebbnrfifHhDYfgasaacH8akY=wiFfYdH8Gipec8Eeeu0xXdbba9frFj0=OqFfea0dXdd9vqai=hGuQ8kuc9pgc9s8qqaq=dirpe0xb9q8qiLsFr0=vr0=vr0dc8meaabaqaciaacaGaaeqabaqabeGadaaakeaacqGGOaakcqWGPbqAdaqhaaWcbaGaemOAaOMaeiilaWIaeGymaedabaGaeiikaGIaemivaqLaeiykaKcaaOGaeiilaWIaeiOla4IaeiOla4IaeiOla4IaeiilaWIaemyAaK2aa0baaSqaaiabdQgaQjabcYcaSiabiIda4aqaaiabcIcaOiabdsfaujabcMcaPaaakiabcMcaPiabg2da9maalaaabaWaaabmaeaacqWG3bWDdaWgaaWcbaGaemiCaahabeaakiabdohaZnaaBaaaleaacqWGWbaCcqGGSaalcqWGQbGAaeqaaaqaaiabdchaWjabg2da9iabigdaXaqaaiabdcfaqbqdcqGHris5aaGcbaWaaabmaeaacqWG3bWDdaWgaaWcbaGaemiCaahabeaaaeaacqWGWbaCcqGH9aqpcqaIXaqmaeaacqWGqbaua0GaeyyeIuoaaaaaaa@5B9D@

Where *w*_*p *_is the weight attributed to the *p*-th template. If the identity between template *p *and the query is idp and the quality of a template (measured as X-ray resolution + R-factor/20, as in [33] – the lower the better) is *q*_*s*_, then it is:

wp=idp3/qp
 MathType@MTEF@5@5@+=feaafiart1ev1aaatCvAUfKttLearuWrP9MDH5MBPbIqV92AaeXatLxBI9gBaebbnrfifHhDYfgasaacH8akY=wiFfYdH8Gipec8Eeeu0xXdbba9frFj0=OqFfea0dXdd9vqai=hGuQ8kuc9pgc9s8qqaq=dirpe0xb9q8qiLsFr0=vr0=vr0dc8meaabaqaciaacaGaaeqabaqabeGadaaakeaacqWG3bWDdaWgaaWcbaGaemiCaahabeaakiabg2da9iabdMgaPjabdsgaKnaaDaaaleaacqWGWbaCaeaacqaIZaWmaaGccqGGVaWlcqWGXbqCdaWgaaWcbaGaemiCaahabeaaaaa@39EE@

Taking the cube of the identity between template and query drastically reduces the contribution of low-similarity templates when good templates are available. For instance a 90% identity template is weighed two orders of magnitude more than a 20% one. In preliminary tests (not shown) this measure performed better than a number of alternatives.

The final two units of *i*_*j *_encode the weighted average coverage and similarity of a column of the template profile as follows:

ij,9(T)=∑p=1Pwpcp∑p=1Pwp
 MathType@MTEF@5@5@+=feaafiart1ev1aaatCvAUfKttLearuWrP9MDH5MBPbIqV92AaeXatLxBI9gBaebbnrfifHhDYfgasaacH8akY=wiFfYdH8Gipec8Eeeu0xXdbba9frFj0=OqFfea0dXdd9vqai=hGuQ8kuc9pgc9s8qqaq=dirpe0xb9q8qiLsFr0=vr0=vr0dc8meaabaqaciaacaGaaeqabaqabeGadaaakeaacqWGPbqAdaqhaaWcbaGaemOAaOMaeiilaWIaeGyoaKdabaGaeiikaGIaemivaqLaeiykaKcaaOGaeyypa0ZaaSaaaeaadaaeWaqaaiabdEha3naaBaaaleaacqWGWbaCaeqaaOGaem4yam2aaSbaaSqaaiabdchaWbqabaaabaGaemiCaaNaeyypa0JaeGymaedabaGaemiuaafaniabggHiLdaakeaadaaeWaqaaiabdEha3naaBaaaleaacqWGWbaCaeqaaaqaaiabdchaWjabg2da9iabigdaXaqaaiabdcfaqbqdcqGHris5aaaaaaa@4B82@

where *c*_*p *_is the coverage of the sequence by template *p *(i.e. the fraction of non-gaps in the alignment), and

ij,10(T)=∑p=1Pwpidp∑p=1Pwp
 MathType@MTEF@5@5@+=feaafiart1ev1aaatCvAUfKttLearuWrP9MDH5MBPbIqV92AaeXatLxBI9gBaebbnrfifHhDYfgasaacH8akY=wiFfYdH8Gipec8Eeeu0xXdbba9frFj0=OqFfea0dXdd9vqai=hGuQ8kuc9pgc9s8qqaq=dirpe0xb9q8qiLsFr0=vr0=vr0dc8meaabaqaciaacaGaaeqabaqabeGadaaakeaacqWGPbqAdaqhaaWcbaGaemOAaOMaeiilaWIaeGymaeJaeGimaadabaGaeiikaGIaemivaqLaeiykaKcaaOGaeyypa0ZaaSaaaeaadaaeWaqaaiabdEha3naaBaaaleaacqWGWbaCaeqaaOGaemyAaKMaemizaq2aaSbaaSqaaiabdchaWbqabaaabaGaemiCaaNaeyypa0JaeGymaedabaGaemiuaafaniabggHiLdaakeaadaaeWaqaaiabdEha3naaBaaaleaacqWGWbaCaeqaaaqaaiabdchaWjabg2da9iabigdaXaqaaiabdcfaqbqdcqGHris5aaaaaaa@4DBD@

It is worth noting how both structural information from templates and the two indices of template quality above are residue-based. For this reason, the case in which only templates covering fragments of a protein exist does not pose a problem for the method – the residues not covered by templates will simply have the section of the input with template information blank, and predictions will be based only on the sequence (and on sequence and template information transmitted by the forward and backwards memory chains). Template information for solvent accessibility is encoded similarly to secondary structure, except that 4 units are adopted to represent average solvent accessibility from PDB-derived templates (4 approximately equal classes). The two units encoding the profile quality are the same as in the secondary structure case. For the comparative experiments without templates, exactly same architectures are adopted, except that the part of the inputs ij(T)
 MathType@MTEF@5@5@+=feaafiart1ev1aaatCvAUfKttLearuWrP9MDH5MBPbIqV92AaeXatLxBI9gBaebbnrfifHhDYfgasaacH8akY=wiFfYdH8Gipec8Eeeu0xXdbba9frFj0=OqFfea0dXdd9vqai=hGuQ8kuc9pgc9s8qqaq=dirpe0xb9q8qiLsFr0=vr0=vr0dc8meaabaqaciaacaGaaeqabaqabeGadaaakeaacqWGPbqAdaqhaaWcbaGaemOAaOgabaGaeiikaGIaemivaqLaeiykaKcaaaaa@3274@ representing the template profile is set to zero.

#### Filtering BRNN

We adopt a second filtering BRNN, similarly to [19]. The network is trained to predict secondary structures (or solvent accessibilities) given first-layer secondary structure (resp. accessibility) predictions. The *i*-th input to this second network includes the first-layer predictions in position *i *augmented by first stage predictions averaged over multiple contiguous windows. That is, if *c*_*j*1_,...*c*_*jm *_are the outputs in position *j *of the first stage network corresponding to estimated probabilities of secondary structure or solvent accessibility *j *being in class *m*, the input to the second stage network in position *j *is the array *I*_*j*_:

Ij=(cj1,...,cjm,∑h=k−p−wk−p+wch1,...,∑h=k−p−wk−p+wchm,⋯∑h=kp−wkp+wch1,...,∑h=kp−wkp+wchm)
 MathType@MTEF@5@5@+=feaafiart1ev1aaatCvAUfKttLearuWrP9MDH5MBPbIqV92AaeXatLxBI9gBaebbnrfifHhDYfgasaacH8akY=wiFfYdH8Gipec8Eeeu0xXdbba9frFj0=OqFfea0dXdd9vqai=hGuQ8kuc9pgc9s8qqaq=dirpe0xb9q8qiLsFr0=vr0=vr0dc8meaabaqaciaacaGaaeqabaqabeGadaaakeaafaqaceabbaaaaeaacqWGjbqsdaWgaaWcbaGaemOAaOgabeaakiabg2da9iabcIcaOiabdogaJnaaBaaaleaacqWGQbGAcqaIXaqmaeqaaOGaeiilaWIaeiOla4IaeiOla4IaeiOla4IaeiilaWIaem4yam2aaSbaaSqaaiabdQgaQjabd2gaTbqabaGccqGGSaalaeaadaaeWbqaaiabdogaJnaaBaaaleaacqWGObaAcqaIXaqmaeqaaOGaeiilaWIaeiOla4IaeiOla4IaeiOla4IaeiilaWYaaabCaeaacqWGJbWydaWgaaWcbaGaemiAaGMaemyBa0gabeaakiabcYcaSaWcbaGaemiAaGMaeyypa0Jaem4AaS2aaSbaaWqaaiabgkHiTiabdchaWbqabaWccqGHsislcqWG3bWDaeaacqWGRbWAdaWgaaadbaGaeyOeI0IaemiCaahabeaaliabgUcaRiabdEha3bqdcqGHris5aaWcbaGaemiAaGMaeyypa0Jaem4AaS2aaSbaaWqaaiabgkHiTiabdchaWbqabaWccqGHsislcqWG3bWDaeaacqWGRbWAdaWgaaadbaGaeyOeI0IaemiCaahabeaaliabgUcaRiabdEha3bqdcqGHris5aaGcbaGaeS47IWeabaWaaabCaeaacqWGJbWydaWgaaWcbaGaemiAaGMaeGymaedabeaakiabcYcaSiabc6caUiabc6caUiabc6caUiabcYcaSmaaqahabaGaem4yam2aaSbaaSqaaiabdIgaOjabd2gaTbqabaaabaGaemiAaGMaeyypa0Jaem4AaS2aaSbaaWqaaiabdchaWbqabaWccqGHsislcqWG3bWDaeaacqWGRbWAdaWgaaadbaGaemiCaahabeaaliabgUcaRiabdEha3bqdcqGHris5aaWcbaGaemiAaGMaeyypa0Jaem4AaS2aaSbaaWqaaiabdchaWbqabaWccqGHsislcqWG3bWDaeaacqWGRbWAdaWgaaadbaGaemiCaahabeaaliabgUcaRiabdEha3bqdcqGHris5aOGaeiykaKcaaaaa@9C76@

where *k*_*f *_= *j *+ *f *(2*w *+ 1), 2*w *+ 1 is the size of the window over which first-stage predictions are averaged and 2*p *+ 1 is the number of windows considered. In the tests we use *w *= 7 and *p *= 7, as in [19]. This means that 15 contiguous, non-overlapping windows of 15 residues each are considered, i.e. first-stage outputs between position *j *- 112 and *j *+ 112, for a total of 225 contiguous residues, are taken into account to generate the input to the filtering network in position *j*. This input contains a total of 16*m *real numbers: *m *representing the *m*-class output of the first stage in position *j*; 15*m *representing the *m*-class outputs of the first-stage *averaged *over each of the 15 windows. *m *is 3 in the case of secondary structure prediction and 4 for (4-class) solvent accessibility prediction.

#### Training, Ensembling

Five two-stage BRNN models are trained independently and ensemble averaged to build the final predictor. Differences among models are introduced by two factors: stochastic elements in the training protocol, such as different initial weights of the networks and different shuffling of the examples; different architecture and number of free parameters of the models. The training strategy is identical to that adopted for Porter [19]: 1000 epochs of training are performed for each model; the learning rate is halved every time we do not observe a reduction of the error for more than 50 epochs. The size and architecture of the models, apart from differences caused by the different number of inputs, is the same as Porter's. The number of free parameter per model ranges between 5,800 and 8,000. The template-based models are only slightly larger (on average 7% more free parameters) than the corresponding *ab initio *ones. Averaging the 5 models' outputs leads to classification performance improvements between 1% and 1.5% over single models. Furthermore a copy of each of the 5 models is saved at regular intervals (100 epochs) during training. Stochastic elements in the training protocol (similar to that described in [14]) guarantee that differences during training are non-trivial. An ensemble of a total of 45 such models yields a further slight improvement over the ensemble of 5 models.

## Authors' contributions

GP suggested the protocol for incorporating templates, designed the overall pipeline, and implemented and tested the secondary structure prediction stage and the web version of the servers. AJMM designed and implemented the stage for homology detection. CM implemented and tested the predictor of solvent accessibility and analysed most of the results. AV contributed to the implementation of all stages and to the analysis of the results. The manuscript was written by GP, AJMM and AV and approved by all authors.
